# Movements and spatial distribution of an endangered fish (*Sciaena umbra*) within a marine protected area

**DOI:** 10.1038/s41598-023-50194-1

**Published:** 2024-02-07

**Authors:** R. Marques, A. Brazo, E. Aspillaga, M. Zimmermann, B. Hereu, G. Saragoni, A. Mercière, R. Crec’Hriou, M. Mercader, M. Verdoit-Jarraya, F. Cadène, Philippe Lenfant

**Affiliations:** 1 Centre de Formation et de Recherche sur les Environnements Méditerranéens, UMR 5110, Université de Perpignan, Via Domitia, 66860 Perpignan, France; 2 Centre de Formation et de Recherche sur les Environnements Méditerranéens, UMR 5110, CNRS, 66860 Perpignan, France; 3 Centre de Recherche sur les Ecosystèmes Marins – Plateforme Intervention et Expertise en Environnement Marin (CREM-IEEM), Impasse du Solarium, 66420 Le Barcares, France; 4 German Center for Marine Biodiversity Research (DZMB), Senckenberg am Meer, Martin-Luther-King Platz 3, 20146 Hamburg, Germany; 5 Institut Mediterrani d’Estudis Avançats, IMEDEA (CSIC-UIB), C/Miquel Marquès 21, 07190 Esporles, Balearic Islands Spain; 6 Department of Evolutionary Biology, Ecology and Environmental Sciences, Institut de Recerca de la Biodiversitat (IRBIO), University of Barcelona (UB), Av. Diagonal 643, 08028 Barcelona, Spain; 7 PSL Research University: EPHE-CNRS-UPVD, UAR 3278 CRIOBE, BP 1013, 98729 Papetoai, Mo’orea French Polynesia; 8 Laboratoire d’Excellence «CORAIL», Papetoai, Moorea French Polynesia; 9 Station Biologique CNRS-Sorbonne Université - Service Observation, Place Georges Teissier CS90074, 29688 Roscoff, France; 10 Marine Eco-Evo-Devo Unit, Okinawa Institute of Science and Technology, Onna-son, Okinawa 904-0495 Japan; 11 Réserve Naturelle Marine de Cerbère Banyuls, 5 Rue Roger David, 66650 Banyuls-sur-Mer, France

**Keywords:** Animal migration, Behavioural ecology, Biodiversity, Conservation biology, Population dynamics

## Abstract

The brown meagre (*Sciaena umbra*) is an endangered species, which requires specific protection measures to ensure its conservation. These measures need to be informed by high-quality scientific knowledge on their space use patterns. Here, we used acoustic telemetry to assess its seasonal movement patterns and habitat use within a marine protected area (MPA). Our results suggested that *S. umbra* is a highly sedentary species (home range < 1.0 km^2^) and, therefore, the MPA is extensive enough to protect the local population. Their population was discretely distributed in two main areas within the MPA, which was likely a result of habitat segregation and density-dependent movements. The temporal variability of their movements further uncovered when and where spawning occurs (mainly, but probably not only, in the fully protected area in June) and indicated that *spillover* of this species is limited but still possible. Overall, we highlight the importance of MPAs in the recovery of *S. umbra*, we advocate the need to perpetuate the current national fishing bans and extend it to other countries in the Mediterranean region, and we emphasize that considering the fine-scale movements of *S. umbra* in future management actions is key to achieving a successful recovery of their populations.

## Introduction

The Mediterranean sea is a well-recognized biodiversity hotspot, but is also under several anthropogenic pressures which threaten its marine biodiversity^1^. Habitat loss, degradation and pollution, overexploitation of marine resources, invasion of species and climate change are considered the most important menaces^1^. In this context, Marine Protected Areas (MPAs) have become key management strategies to protect marine habitats and biodiversity^2^. Indeed, to successfully attain biodiversity conservation at a global level, the International Union for Conservation of Nature (IUCN) proposed to protect 30% of the world’s marine areas by 2030^3^. This supports the creation of new MPAs or increasing the size of currently existing ones if they are proven to provide effective protection against biodiversity loss and directly or indirectly support ecosystem services^2^. MPAs are widely recognized as important tools for fisheries management and the tourism-based blue economy, where higher abundance, biomass and diversity of marine organisms attract divers and support the local recreational and commercial small fisheries^4–6^.

MPAs are areas where anthropogenic activities are highly regulated, frequently under multiple protection levels in order to balance conservation and exploitation interests^5^. This often includes fully protected areas (FPAs, or no-take zones), where marine harvesting is not allowed, and partially protected areas (PPAs) where some activities (including fishing) are permitted under specific regulation. One of the main benefits of MPAs is the enhancement of exploitable biomass of fish in adjacent non-protected areas^4,7,8^. The high biomass and abundance of fishes within FPAs subsidize PPAs and non-protected areas with fish adults and early stages (e.g. eggs, larvae and juveniles), in a process known as “spillover”^9^. However, the spillover effect largely depends on MPAs characteristics (size, shape, location, age, levels of protection, habitat, etc.)^5^. A stronger spillover effect is expected in older and larger MPAs with multiple protection zones (i.e. FPA surrounded by a PPA)^5,10^ due to an increase in the reproductive capacity of adult fish populations within FPAs, which will later provide fish biomass to the surrounding areas^11,12^. The protection of larger fish individuals within a FPA will further endorse the spillover since they exhibit disproportionately higher reproductive output than the smaller fish outside MPAs^12^. The spillover also depends on the fish species, since it is conditioned by their mobility, intra-specific behavior and habitat preference. This will influence their direct biomass export from the MPA, but also their reproductive efficiency and the ultimate dissemination of young fish stages^5,7,10,13^. Therefore, to protect biodiversity and increase the spillover effect of an MPA, a full understanding of species specific spatial and temporal movements within the protected areas is required. This is particularly important for endangered species.

The brown meagre (*Sciaena umbra*) is a threatened species with high commercial value, currently classified as vulnerable in the Mediterranean region^14,15^. Heavy exploitation of this species has caused a drastic reductions of their populations and MPAs are now considered the most effective management tools to help in their recovery^15^. Efforts have been made to protect *S. umbra* populations, particularly in France, where a moratorium ban fishing this species over the entire national Mediterranean coast until 2023 (Regional order R93-2018-12-20-002, 20 December 2018). However, its highly accessible habitats, its gregarious character, and its peaceful behavior, make it an easy and still vulnerable target for artisanal and recreational fisheries^15–17^, further underlining the role of MPAs in the recovery of *S. umbra* abundance and biomass within and outside of the protected areas^7,18^. Yet, knowledge on the spatial activity of this species is still required to sustain and improve management strategies. For example, a recent study on the vertical movements of *S. umbra* in an MPA^19^ revealed that this species presents a seasonal pattern of bathymetric distribution: part of the population inhabits shallow waters all year round, while the remaining individuals migrate from deeper waters during the cold months to shallow waters in warmer months, where the whole population aggregate. This spatio-temporal variability was suggested to be associated with the reproductive and foraging activities in warm and cold periods, respectively. However, very little is still known regarding the temporal variability of their horizontal habitat utilization in MPAs. Resolving these movements provides critical information on whether the protected areas are large enough to encompass their home range and spawning locations, and if MPAs have proper conditions for an effective reproductive activity of the species^20^. This ecological knowledge would increase our understanding on the efficiency of the marine reserves and could guide further protection measures to boost the recovery of *S. umbra* populations^21^.

Passive acoustic telemetry has been increasingly used by researchers to study fish movements by limiting human interference^22,23^. This technique uses networks of acoustic receivers and small acoustic transmitters implanted in fish, which continuously monitor their movements at high spatial and temporal resolutions, to determine key behavioral aspects such as the spatial and temporal mobility^24–26^, site fidelity^27,28^ and habitat use^24,29,30^. Several studies have successfully used passive acoustic telemetry to elucidate the movement patterns of fish in MPAs^26,31–33^, including in the Mediterranean Sea^24,30,34–36^. However, very few studies have been carried out on the horizontal movements of the brown meagre^37–39^ and those were performed on few individuals and over short periods of less than 1 year.

In the present study, we used passive acoustic telemetry to resolve the seasonal spatial movement patterns of *S. umbra* and their fidelity to the *Réserve Naturelle Marine de Cerbère-Banyuls* (RNMBC), to better understand when and where critical periods of their life cycle occur. Our final goal was to provide sustained ecological information to support adequate protection measures to achieve an effective recovery of the *S. umbra* populations.

## Material and methods

### Study site

**Figure 1:**
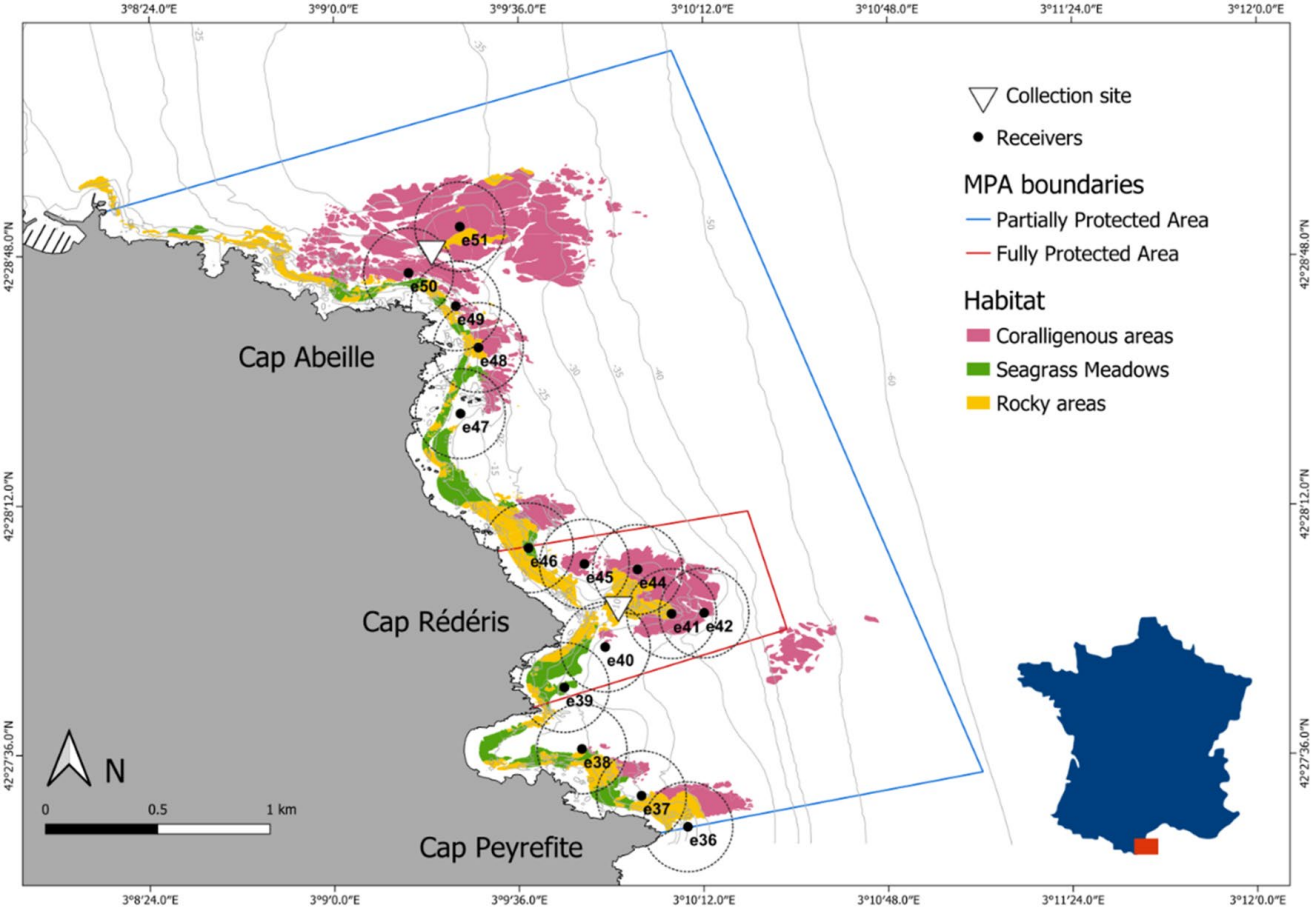
Study site and the acoustic receiver’s array at the RNMCB (*Réserve Naturelle Marine de Cerbère-Banyuls*).

The spatial distribution and the temporal variability in the movements of the brown meagre (*Sciaena umbra*) were investigated in the *Réserve Naturelle Marine de Cerbère-Banyuls* (RNMCB, Fig. 1). The RNMCB is a French marine protected area located in the North-Western Mediterranean Sea. Established in 1974, it is one of the oldest marine protected areas in the Mediterranean and it greatly contributes to the socio-economic development of the region, in particular for recreational diving and artisanal fisheries^40,41^. Since 2011, the reserve is part of the larger (4010 km^2^) marine natural park of the Gulf of Lion (https://www.parc-marin-golfe-lion.fr/), where human activities are virtually not restricted, but it allows a consistent management of the local biodiversity at larger space scales. Covering 6.5 km of coastline and up to 1.5 nautical miles offshore, the RNMCB has a total area of 650 ha^42^ and it comprises two areas with different levels of protection: the fully protected area (FPA), with 65 ha nearby Cap Rédéris, and the Partially Protected Area (PPA), with 585 ha surrounding the FPA. In the FPA only recreational navigation, surface swimming and scientific diving are authorized, while in the PPA recreational activities, such as scuba diving, boat circulation and daytime angling are allowed. Thanks to its ecological health and habitat diversity (rock and boulder bottoms, coralligenous outcrops, and seagrass meadows), the reserve provides a suitable environment for the brown meagre^15,42^.

### Fish sampling

Twenty brown meagre were tagged and monitored within the RNMCB between June 2016 and December 2018. Fish were tagged and released at two collection sites: close to Cap Rédéris, within the FPA and close to Cap Abeille in the PPA (Table 1, Fig. 1). Sampling was performed at three different periods (June–July 2016, October 2016 and June–August 2017), using dip nets (CAPERLAN, diameter 50 cm, mesh 3 mm), during the night and at a maximum depth of 10 m^19,36,43^. Captured individuals were brought slowly to the surface to avoid decompression injuries and stress^43^. Once in the boat, the individuals were transferred to an anesthesia tank of 100 L, filled with in situ seawater and 15 mL of anesthetic solution (70% alcohol and 30% clove oil).

**Table 1:** Summary of the monitoring information of the tagged *S. umbra* individuals.

Fish ID	Total length (cm)	Capture site	Capture date	Last detection	Total detections	Tracking period (d)	Days detected	RI	Days in FPA	RI in FPA	Depth range (m)	Core area (km^2^)	Home range (km^2^)	Depth utilisation group
419	33	Cap Abeille (PPA)	08/06/2016	24/10/2017	39,904	504	364	0.72	0	0	0.3–20.9	0.07	0.31	DS
424	37		08/06/2016	21/06/2017	19,030	379	316	0.83	1	0	2.3–27.7	0.15	0.57	DS
429	39		08/06/2016	09/06/2017	12,350	367	229	0.62	0	0	0–19.6	0.08	0.51	SS
455	37		21/07/2017	08/12/2018	63,186	506	504	1	2	0	2.7–22.1	0.08	0.40	DS
456*	38		21/07/2017	10/08/2017	2150	21	21	1	0	0	6.4–19.3	NA	NA	NA
462*	45		22/08/2017	29/11/2017	2623	100	59	0.59	0	0	8.6–32	NA	NA	NA
3031	36		16/06/2017	03/11/2018	57,262	506	497	0.98	3	0.01	2.3–27.3	0.09	0.42	DS
3037	35		20/07/2017	15/06/2018	66,705	365	339	0.93	0	0	3.7–76.1	0.10	0.50	DS
3038	34		16/06/2017	03/11/2018	43,683	506	492	0.97	2	0	1.8–21.3	0.08	0.39	SS
3039	56		11/07/2016	04/11/2017	39,932	482	279	0.58	0	0	4.3–31.5	0.09	0.46	DS
457	42	Cap Rédéris (FPA)	03/08/2017	21/12/2018	51,455	506	506	1	505	1	1.6–15.2	0.17	0.81	SS
458	47		03/08/2017	21/12/2018	164,591	506	506	1	506	1	5–35.6	0.17	0.66	DS
459	36		21/06/2017	08/11/2018	45,532	506	460	0.91	459	0.91	0.8–18.2	0.17	0.69	SS
460	43		03/08/2017	21/12/2018	66,404	506	505	1	88	0.17	4.4–19.3	0.09	0.82	SS
461	55		03/08/2017	21/12/2018	70,416	506	506	1	505	1	4.9–20.7	0.19	0.76	SS
3032	35		04/10/2016	11/10/2017	105,739	373	371	0.99	371	0.99	7.2–18.2	0.09	0.53	SS
3033	35		04/10/2016	21/02/2018	169,896	506	488	0.96	488	0.96	4.4–38.4	0.19	0.79	DS
3034	33		04/10/2016	28/09/2017	36,868	360	355	0.99	355	0.99	0.8–18.8	0.10	0.63	SS
3035*	32		04/10/2016	14/10/2016	2415	11	11	1	11	1	10–38.2	NA	NA	NA
3036	46		21/06/2017	08/11/2018	43,946	506	505	1	505	1	3.8–20.8	0.08	0.52	SS

Depths are mean daily depths. Depth utilisation group was defined by Brazo et al.^19^: individuals that remained in shallow waters all year round (SS shallow/shallow) and those that presented a seasonal shift from deep to shallow waters during warmer months (DS deep/shallow).

*RI* residence index, *FPA* fully protected area, *PPA* partially protected area.

*Fish not included in the analysis.

### Fish tagging

Fish were measured, placed inverted, and tagged with V13P-1H acoustic transmitters (VEMCO, Nova Scotia, Canada, dimensions: 45 mm long by 13 mm in diameter). Transmitters were equipped with a pressure sensor and were programmed to emit signals every ca. 2 min (± 10 s). Only adult individuals with more than 30 cm of total length were tagged to guarantee that the weight of the transmitter did not exceed 2.6% of the fish weigh, ensuring the welfare of the fish^24^. The transmitters were placed in the coelomic cavity of the fish after a standard surgical procedure^43,44^. Tagging was conducted by trained and licensed scientists working under the authority and approval of the *Certificat d’experimenter sur les animaux vertébrés vivants* (experimental live animal certificate) number 66.0801 (Elisabeth Faliex) of the CEFREM, University of Perpignan. The departmental council of the Pyrénées Orientales and the scientific council of the *Réserve Naturelle Marine de Cerbère-Banyuls* granted us the authorizations to capture, mark and release the brown meagre individuals within the MPA. The manipulation of live vertebrate animals was also approved by the Ethics Committee on Animal Experimentation of the University of Barcelona (*Comitè d’Ètica d’Experimentació Animal de la Universitat de Barcelona*) and accredited by the Government of Catalonia (*Generalitat de Catalunya, Departament de Territori i Sostenibilitat, Direcció General de Polítiques Ambientals i Medi Natural*), under the license no 11218, granted to Bernat Hereu. All operations were performed ensuring the minimum stress of the fish and were all in compliance with the regulations expressed in the aforementioned license and the ARRIVE guidelines^45^. The sex of the individuals could not be determined since *S. umbra* lacks sexual dimorphism^46^. However, all individuals were bigger than the reported size at first maturity (20–30 cm^46,47^), suggesting that they were all mature adults. The individuals were monitored after release by scuba divers to ensure total recovery after the surgery.

### Network of the acoustic receivers

The network of the acoustic receivers (17 receivers, VR2W, VEMCO, Novia Scotia, Canada) was composed of nine receivers within the FPA and eight in the PPA (Fig. 1). The receivers located in the FPA were located at ca. 8 m depth, moored with sub-surface buoys and oriented towards the bottom, while the receivers at the PPA were fixed close to the bottom, oriented towards the surface, in order to minimize potential equipment loss or damage. The receivers have a battery life of about 6 months and a detection range of about 200 m radius, however, local abiotic and biotic factors can reduce the detection performance of acoustic signals^22,48^.

### Data analysis

Acoustic telemetry data were retrieved from the receivers and gathered in a single integrated database using the VUE software (VEMCO, Nova Scotia, Canada). Data was first scrutinized to eliminate spurious detections such as unique detections within 24 h or duplicate detections in different receivers.

For each individual we calculated 5 movement metrics to assess *S. umbra* behavior. The residence index (RI) was calculated for each individual as the proportion of days with detections within our receivers array in relation to the total number of days monitored^37^. The same index was also calculated in regards to the proportion of days spent within the FPA (RI in FPA).

The daily core area (CA) and home range (HR) sizes were calculated by applying a Brownian bridge movement model (BBMM^49,50^) to the detection data. BBMM was used to compute the utilization distribution (UD) of each individual over time (daily and monthly), i.e. the probability distribution of the individual’s use of space, using the BBMM package (v.3.0) for R^51^. CA and HR were calculated as the minimum areas encompassing the 50% and 95% of the UD estimate volumes, respectively, using the package adehabitatHR (v.0.4.14) for R^52^.

The monthly UDs of each individual were used to calculate the percentage of fish with partially or fully overlapping CAs (OCA) over time. To avoid the influence of the low number of tracked individuals per month, particularly at the beginning (June to September 2016) and end (December 2018) of the study period, only months with at least 35% (6 out of 17) of fish simultaneously tracked were considered.

The Daily Distance (DD) performed per each individual was calculated as the sum of the distance between two consecutive detections, based on the half linear distance between receivers. We recognize the existing bias between DD and the real distance performed by the fish due to the fixed distance between receivers and the large positioning uncertainty caused by the acoustic range. Therefore, this measure was not examined quantitatively as true distances but considered for intraspecific comparisons of their movements. The DD was also compared with the daily number of unique receivers visited by each individual.

Generalized Additive Mixed Models (GAMM) were used with the mgcv package (function gamm())^53^ for R, in order to assess (1) the existence of seasonality and temporal trends of HR and DD and (2) the effect of the explanatory variables on HR and DD. GAMMs use a sum of smooth functions to model the effect of covariates, allowing non-linear relationships, the introduction of temporal correlation and various heterogeneity patterns in the model^54^. To assess the existence of temporal trends and the effect of the seasonality, HR and DD (*Response*) were modelled as a function of collection site (*Cs*) and time, using Julian day (*Julian day*_*s*_) to account for seasonality, date (*Date*_*s*_, as a numeric variable) to account for temporal trends, with one intercept (α) and fish ID as a random effect (*a*_i_):

$$Response_{is} = \alpha + a_{i} + Cs_{is} + f\left( {Julian \,day_{s} } \right) + f\left( {Date_{s} } \right) + \epsilon_{is}$$

To determine the effect of biotic and abiotic pressures on the observed HR and DD (*Response*), several variables were used as covariates: seawater temperature (*T*, °C) at 15 m depth, thermocline depth (*Thermo*, m), wind speed (*Ws*, m s^−1^), wind direction (*Wd*, °), wave height (*Wh*, m), fish total length size (*TL*, cm), depth utilization group (*Dgroup*) and capture site (*Cs*), with one intercept (α) and fish ID as a random effect (*a*_i_):

$$Response_{i} = \alpha + a_{i} + f\left( {T_{i} } \right) + f\left( {Ws_{i} } \right) + f\left( {Wd_{i} } \right) + f\left( {Wh_{i} } \right) + f\left( {TL_{i} } \right) + Cs_{i} + Thermo_{i} + Dgroup_{i} + \epsilon_{i} .$$

Seawater temperature data were provided by the MPA managers (T-mednet Interreg MED project, https://t-mednet.org/) recovered from several thermometers placed within the MPA (8 thermometers placed between 5 and 40 m depth in intervals of 5 m). The depth of the thermocline, when present, was calculated using a four-parameter nonlinear regression, fitted to the vertical profile of temperature^55,56^. Using the output of the model, the temperature was estimated for every 0.1 m depth and the mid-depth of the thermocline was defined as the point at which the first derivative of the model corresponded to the fastest range of temperature change. Thermocline depth was only calculated for the profiles where the total temperature difference between the surface and the deepest measures was higher than 3 °C^55^. Individuals were classified in depth utilization groups that represented the typical seasonal pattern of vertical distribution defined by Brazo et al.^19^. SS (shallow/shallow) group represented the individuals that spend all year round in shallow waters, while the DS (deep/shallow) group represented the individuals that spend cold months in deeper waters and warmer months in shallow waters.

Before modeling, pairwise Spearman correlations were calculated to assess covariates collinearity. Covariates with > 0.5 correlation with any other covariate were not included in the model. Wind direction and wave height were, therefore, eliminated since they were correlated with wind speed (0.67 and 0.52, respectively). The thermocline was only present during some months of the year and it was correlated with seawater temperature (0.79). Therefore, the effect of thermocline was included as a factor variable of presence/absence. In all models, we tested the need to include a random effect, a variance structure and a temporal autocorrelation structure, to account for individual variability, lack of homogeneity and lack of independent observations due to temporal autocorrelation, respectively. ACF (autocorrelation function) plots were used to confirm the existence of temporal autocorrelation in the response variable^57^. AIC (Akaike information criterion) was used to select the model with or without random effect, the best variance structures and the most appropriate autocorrelation structure (autoregressive moving average models, ARMA), tested with up to two terms for each the autoregressive and the moving average parameters^13,58^. Standardized residuals were plotted against fitted and observed values to check for homogeneity^54^. In all cases, and based on the AIC model selection, Fish ID was included as a random effect to account for individual variability, and ARMA (2,2) was the best autocorrelation structure identified for all models. A variance structure was added to all models allowing heterogeneity within Fish ID along time, for models testing seasonality and trend; along wind speed, for models testing the influence of environmental data on HR; and along temperature, for models testing the influence of environmental data on DD. After selecting the best random effect, temporal autocorrelation and variance structure, we used backwards selection by eliminating the least significant covariate until all terms were significant to achieve the best fixed terms for the model. The best supported model was selected based on AIC and parsimony^54^. Data analysis was performed in R^59^ and all maps were performed in QGIS^60^, source CEFREM (Centre de Recherche sur les Ecosystèmes Marins).

## Results

### Overview

During the whole study period, a total of 1,104,087 valid detections were recorded from the twenty *S. umbra* individuals tagged in the RNMBC (Table 1). Fishes were tracked for at least 12 months (360–506 days, depending on the fish, Table 1). This excludes three individuals that were not included in the analysis: two individuals (3035 and 456) disappeared from the detection array after 11 and 21 days, respectively. The individual 3035, collected in the FPA, was, however, detected in southern coastal areas (Cap de Creus) at about 21 km south from its collection site 10 days after leaving our study area and, sporadically, 14 and 16 months later. The individual 462 was only tracked for about 3 months (100 days) and was, therefore, also eliminated from the analysis. The remaining individuals inhabited the study area for the whole tracking period. The total length of the individuals ranged between 33 and 55 cm and they inhabited areas with maximum depth of 76.1 m (Table 1).

### Residency and space use

All the analysed *S. umbra* individuals showed high site fidelity to the RNMCB (mean RI ± SE of 0.91 ± 0.03, Table 1). Fish collected at the FPA (Cap Rédéris), presented higher RI values (0.91–1) than those captured in the PPA (Cap Abeille) (0.58–1; Wilcoxon test, W = 11.5, *p* value = 0.02). Both fish size and tracking period did not affect the overall residence index (LM, R^2^ = 0.03 and 0.06, *p* values = 0.52 and 0.35, respectively) or individual home range (LM, R^2^ = 0.08 and 0.02, *p* values = 0.26 and 0.62, respectively).

**Figure 2:**
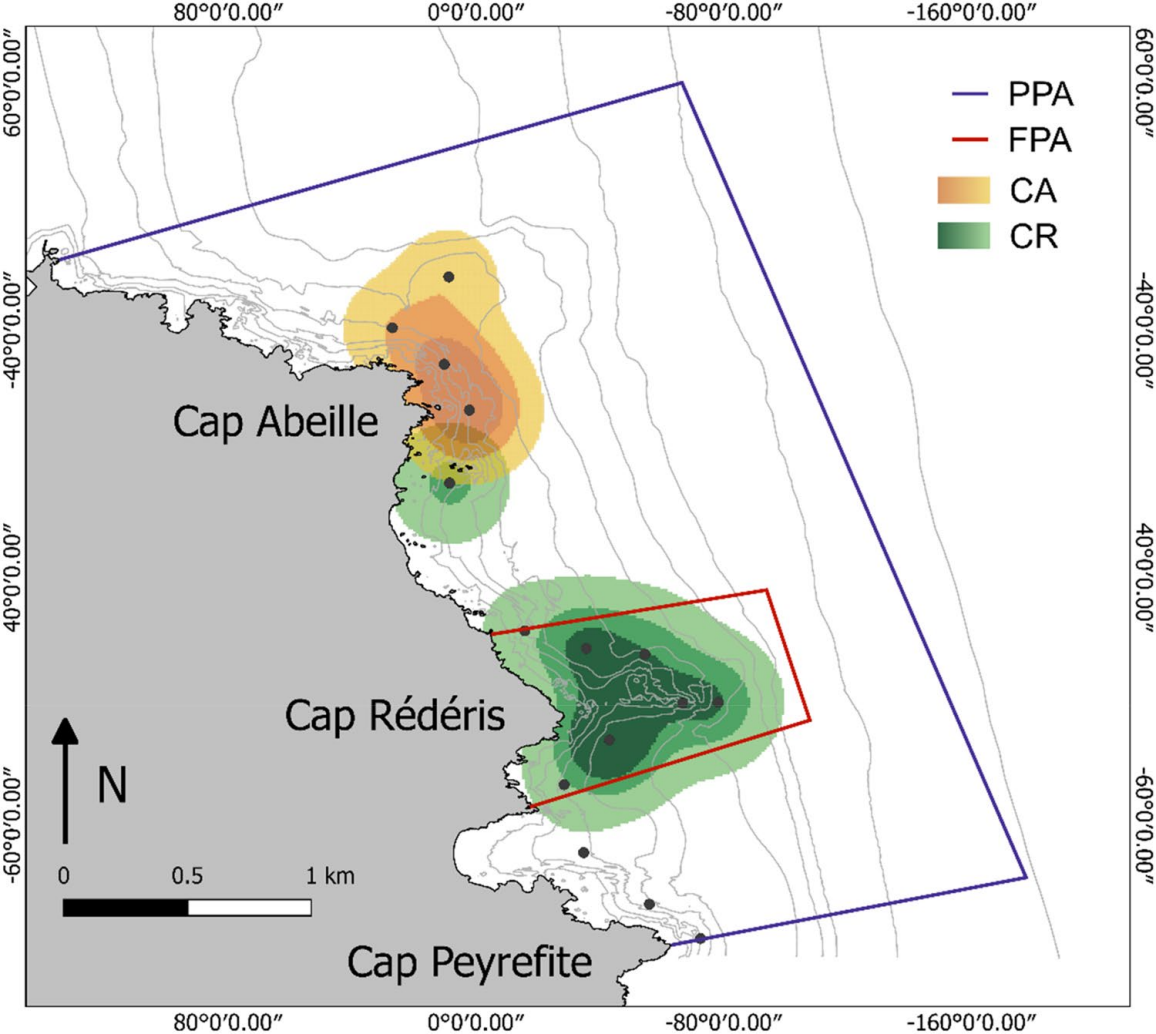
Mean spatial utilization distribution estimates (UD) of *S. umbra* individuals at the *Réserve Naturelle Marine de Cerbère-Banyuls*, for each capture site: in yellows, individuals captured in the PPA (CA: Cap Abeille) and in greens, individuals captured in the FPA (CR: Cap Rédéris). To each capture site, different gradient colours represent, from inside out, the areas covering 50% (core area), 75% and 95% (home range) of the UD volume, computed by the Brownian Bridge Movement Model. *FPA* fully protected area, *FPA* partially protected area. Black dots represent the position of the receivers.

All fish revealed high levels of site fidelity. *Sciaena umbra* individuals inhabited small areas close to their collection site (Fig. 2) with individual core areas ranging from 0.08 to 0.19 km^2^ and 0.07 to 0.15 km^2^ for those captured in the FPA and the PPA, respectively (Table 1). Their home range extended to a maximum of 0.82 and 0.57 km^2^, respectively. Both the CA and the HR significantly differed between individuals collected in different areas (Wilcoxon test, W = 12, *p* value = 0.02 and W = 2, *p* value < 0.01, respectively).

**Figure 3:**
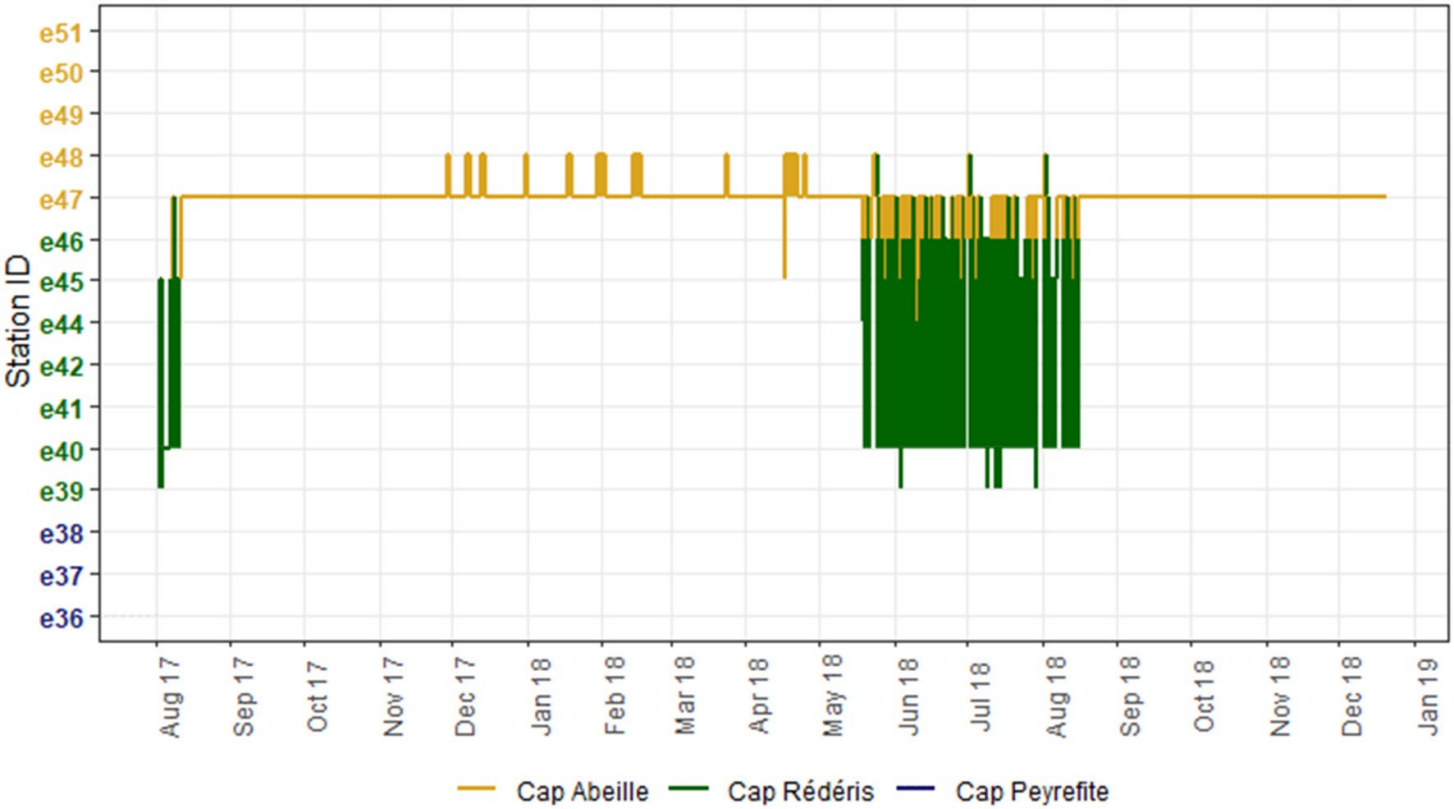
Spatial movements of the individual 460 over time within the study area. The continuous line represents movements between each receiver (Station ID). Colours represent three different zones within the study area: close to Cap Abeille (PPA) in the northern area (yellow), close to Cap Rédéris, within the FPA (green), and close to Cap Peyrefite in the southern area, outside FPA (blue). See Fig. 1 for the exact position of receivers.

Only five individuals (3031, 424, 3038, 455, 460) performed excursions from their collection sites to the adjacent areas. Among those, only one was collected in the FPA (460), while the remaining fish were collected in the PPA and performed excursions to the FPA (or further south to Cap Peyrefite: fish 3038). Those individuals remained in FPA for 1–3 days and then returned to their original area. These excursions were all performed at the same period of the year (between 29 May and 17 June from different years). For two of those fish (3031 and 424) these excursions were done on the next days following their tagging, while for the remaining individuals (3038, 455) these excursions were performed about 12 and 10 months after tagging, respectively. The fish 460 revealed a particular seasonal movement pattern (Fig. 3): it was collected in early August 2017 in the FPA but, after 8 days, it travelled to the PPA (station e47), where it remained until spring 2018, and explains the location of its HR in this area (Fig. 3). From mid-May to mid-August 2018 (89 days), this individual frequently travelled between station e47 and the FPA area, being detected within the FPA for 88% of this time.

**Figure 4:**
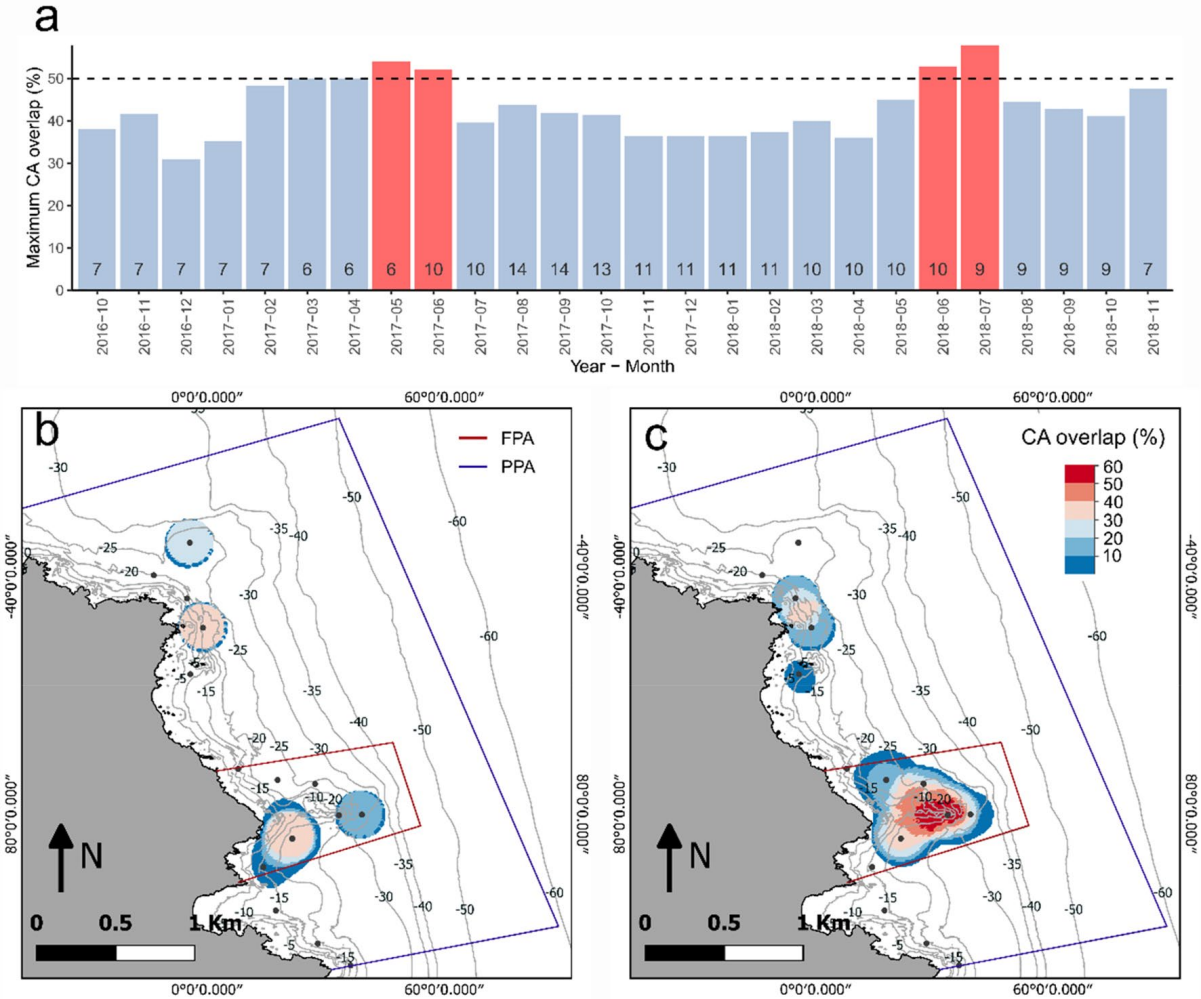
Overlap of individual core areas (CA overlap) of* S. umbra*, i.e. the percentage of individuals sharing, at least partially, the spatial distribution of their CA. In (**a**) bars represent the maximum registered percentage of overlap, at any point of the receiver’s array. Red bars represent months with CA overlap above 50%. Numbers within bars are the numbers of individuals simultaneously tracked at that particular month. Only complete months with at least 35% (6 out of 17) individuals simultaneously tracked were considered. In (**b**) and (**c**) two examples are shown with minimum and maximum levels of CA overlap (December 2016 and July 2018, respectively). *FPA* fully protected area, *FPA* partially protected area.

The clear separation of the spatial utilization distribution of *S. umbra* collected in different zones is further highlighted by the limited shared areas among individuals. Less than 50% of the fish shared their spatial distribution (i.e. CA) over time (Fig. 4a and b), except for 4 months (May, June 2017, and June, July 2018), when 54–58% of the individuals shared, at least partially, their CA. These higher levels of individual’s spatial overlap occurred within the FPA, especially around receivers e41 and e44, where the habitat is composed of rocky bottoms and coralligenous outcrops, at 5–20 m depth (Fig. 4c).

### Temporal variability of home range and daily distances

**Figure 5:**
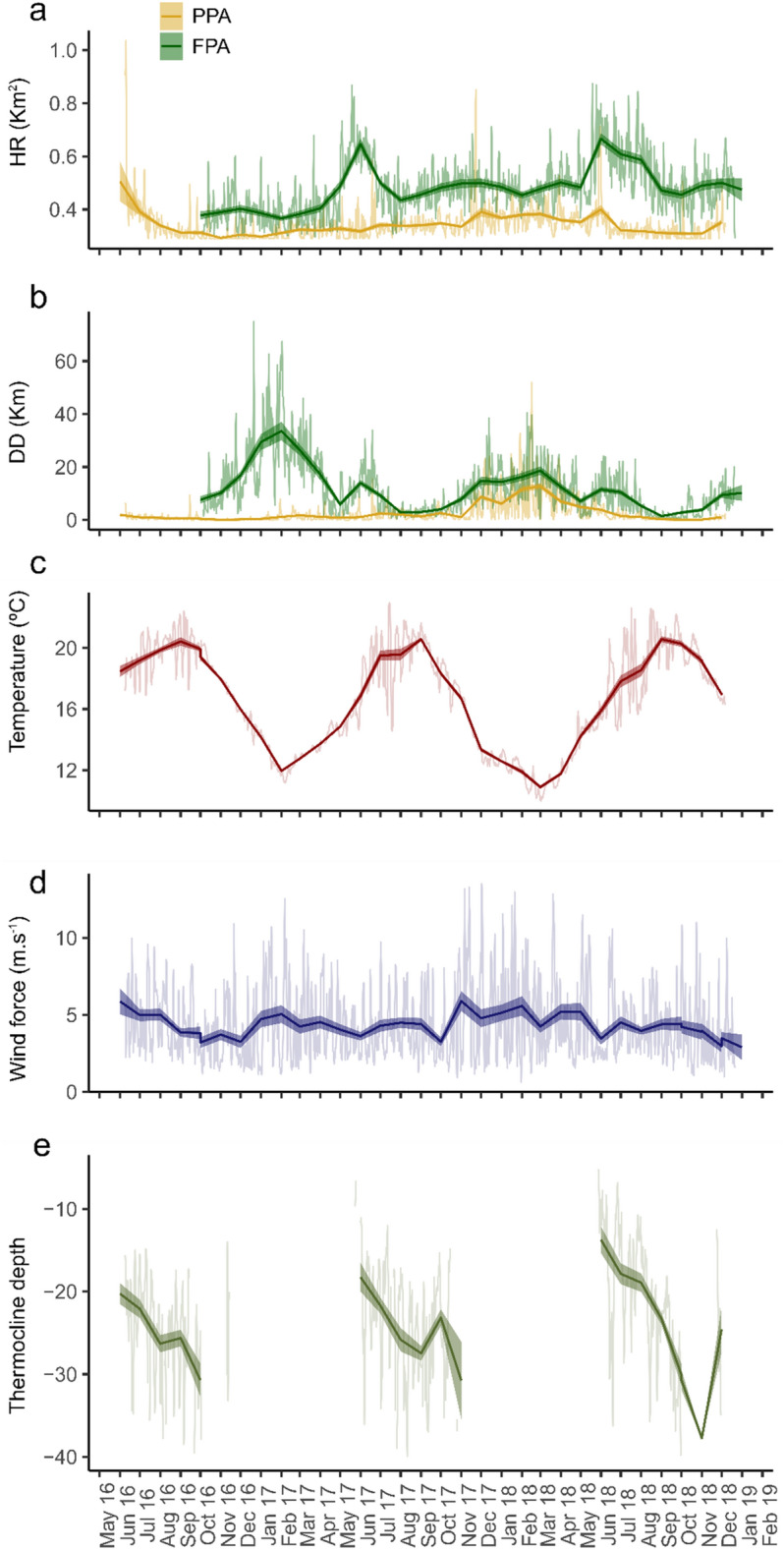
Temporal variability of (**a**) home range (HR), (**b**) daily distances (DD) of the individuals collected in the PPA and FPA, (**c**) temperature (ºC) at 15 m depth, (**d**) wind speed (m s^−1^) and (**e**) depth of the thermocline. Light colour lines are daily values and dark bold lines are mean monthly values (± SE).

**Figure 6:**
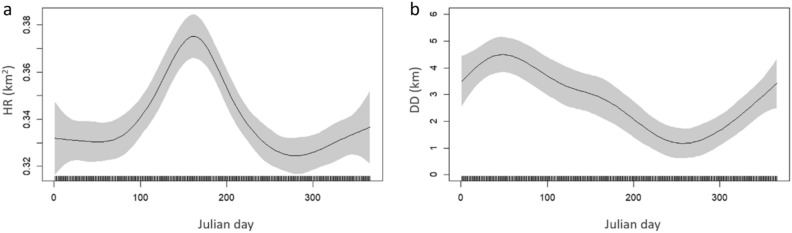
Partial effects of Julian day (test of seasonality) of GAMM models on HR (**a**) and DD (**b**). Shaded areas represent the 95% confidence interval for the mean shape of the effect.

Home range size (HR) and daily distances (DD) varied over time and between individuals collected at different sites (Fig. 5a and b). The running monthly mean HR during the whole study period of the individuals captured in the FPA (Fig. 5a), showed low levels (0.37 ± 0.01 to 0.5 ± 0.01 km^2^), except in June 2017 and June to August 2018, when it reached a maximum of 0.65 ± 0.02 and 0.67 ± 0.02 km^2^, respectively. Individually, the maximum daily HR of 3.9 km^2^ was registered for individual 457 in January 2018. The running monthly mean HR of the individuals captured in the PPA remained below 0.4 km^2^ during the whole study period, except at the beginning of the study period, in June 2016, when it reached 0.51 ± 0.08 km^2^ (Fig. 5a). Still, several peaks of daily HR were observed, which overcome 0.6 km^2^ and occurred in November 2017 and in May 2018. These peaks were likely affected by the aforementioned excursions of some particular individuals captured in the PPA (3031, 3038, 424, and 455), which showed maximum daily HR peaks between 1.03 and 2.22 km^2^ during these months. The results from GAMM models confirmed the significant effect of the collection site on HR (GAMM, t-value = 5.37, *p* value < 0.01) and revealed the existence of a significant seasonality pattern of HR, i.e. a significant effect of Julian day (GAMM, F = 11.14, *p* value < 0.01). The partial effect of Julian day showed higher values in June (Julian day between 152 and 181, Fig. 6). The temporal trend was not significant and not retained in the final model.

While HR increased in June, the daily distances performed by *S. umbra* increased in winter months (Fig. 5b). Individuals collected in the FPA travelled larger distances per day over the entire study period than those collected in the PPA, with an overall mean DD of 10.8 ± 0.27 and 3.3 ± 0.12 km, respectively (Wilcoxon test, W = 3,289,759, *p* value < 0.01). The running monthly mean DD for individuals captured in the FPA revealed lower values between August and October 2017 (< 4 km) and two main annual peaks: the highest peak in February 2017 and March 2018 (34.0 ± 3.57 and 19.1 ± 1.70 km, respectively) and a second annual peak, with lower values though, in June (14.8 ± 1.29 and 11.7 ± 1.03 km, in 2017 and 2018, respectively Fig. 5b). The temporal variability of DD was similar for all individuals in 2018, but in 2017 the individuals collected in the PPA revealed constant lower DD values until November 2017 (< 2.6 km). Maximum values for those fishes were recorded in March 2018 (12.9 ± 1.17 km). Interestingly, the peaks of DD, which overcome 100 km, were not registered for the fish that performed long excursions between the two collection sites, but instead by individuals with relatively small home ranges (3033, 3034 and 458, see Table 1). The GAMM models confirmed the significant effect of the capture site on the DD (GAMM, t-value = 4.93, *p* value < 0.01) and revealed the existence of a significant seasonality and trend of DD, i.e. a significant effect of Julian day and date (GAMM, F = 6.61 and F = 6.66, *p* values < 0.01, respectively). The partial effect of Julian day showed higher values in February (Julian day between 32 and 59) and lower values in September (Julian day between 244 and 273, Fig. 6). Although the temporal trend was significant, with a non-linear pattern over time, it was not further considered since our data comprised only 2.5 years.

Several environmental parameters were considered as possible drivers of the observed temporal variability of HR and DD (Fig. 5c–e). Temperature at 15 m depth followed the typical trend observed in temperate areas (Fig. 5c), with higher values between July and October (> 19 °C) and minimum values in February 2017 and March 2018 (12.0 ± 0.08 and 10.9 ± 0.11 °C, respectively). The wind speed was very variable over the entire study period (Fig. 5d), with minimum and maximum monthly mean values registered in December 2018 (2.97 ± 0.41 m s^−1^) and in November 2017 (5.90 ± 0.65 m s^−1^), respectively. The thermocline was only present when the temperature was high (between June and October) and ranged from 5.21 to 39.94 m depth (daily values, Fig. 5e).

**Figure 7:**
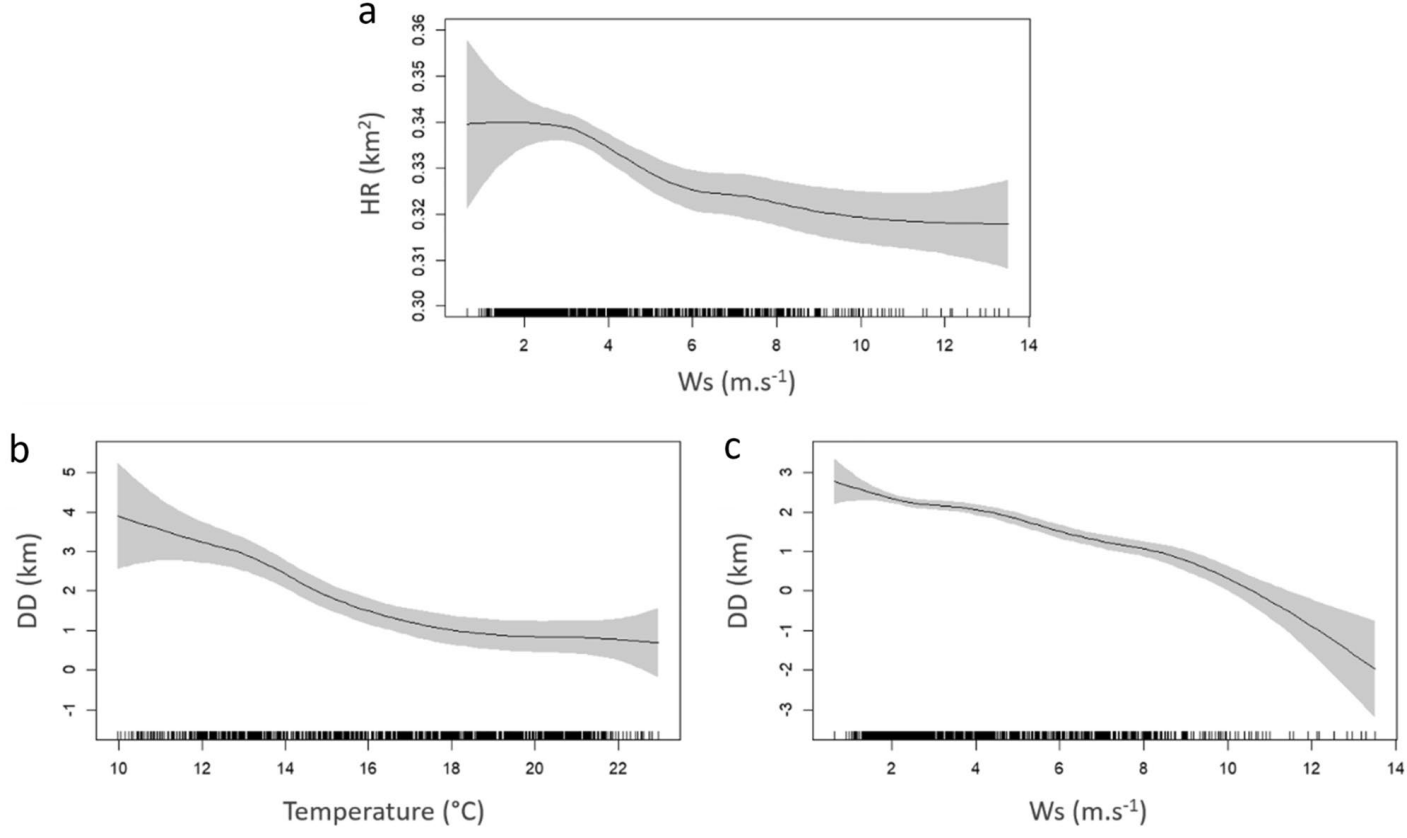
Partial effects of the significant fixed terms from the best supported GAMM model on HR (**a**) and DD (**b**,**c**). Shaded areas represent 95% confidence interval for the mean shape of the effect.

Among the factors included in the GAMM model to assess the effect of environmental data on HR and DD (Table 2), the capture site was consistently kept in the final model, confirming the previously described differences in the movements between *S. umbra* individuals collected at different sites. The best-supported model for HR included the fixed effects of temperature, wind speed, fish size, thermocline and capture site, although only the capture site and wind speed significantly affected HR. As previously described, the HR of fish collected in the FPA was significantly higher than those captured in the PPA, with an estimated parameter of 0.1 km^2^ (GAMM, t-value = 4.09, *p* value < 0.01), while wind speed had a significant negative effect (GAMM, F = 5.35, *p* value < 0.01, Fig. 7a), particularly at wind speed higher than ca. 4 m s^−1^. For DD, the best-supported model indicated that capture site, temperature and wind speed were all important explaining its temporal variability (Table 2). Like for HR, fish collected in the FPA travelled longer distances per day, with an estimated difference of 4.7 km (GAMM, t-value = 2.68, *p* value = 0.007). Temperature and wind speed significantly affected *S. umbra* DD (GAMM, F = 6.44 and 35.3, *p* values < 0.01, respectively). The effect of temperature and wind speed were negative, suggesting that *S. umbra* individuals perform larger distances at temperatures < 14 °C (Fig. 7b) and are highly limited by winds exceeding 10 m s^−1^ (Fig. 7c).

**Table 2:** Results from model selection of fixed terms for daily home range (HR) and daily distances (DD).

Model	df	AIC	ΔAIC	LogLik
Daily home range (HR)				
HR ~ s(T) + s(Ws) + s(TL) + Thermo + Cs + Dgroup	33	− 11,707.2	28.3	5886.6
**HR ~ s(T) + s(Ws) + s(TL) + Thermo + Cs**	**32**	**−** **11,735.5**	**0.0**	**5899.8**
HR ~ s(T) + s(Ws) + s(TL) + Cs	31	− 11,634.0	101.5	5848.0
HR ~ s(Ws) + s(TL) + Cs	29	− 11,723.2	12.4	5890.6
HR ~ s(Ws) + Cs	27	− 11,661.9	73.6	5858.0
HR ~ s(Ws)	26	− 11,669.7	65.9	5860.8
Daily distances (DD)				
DD ~ s(T) + s(Ws) + s(TL) + Thermo + Cs + Dgroup	32	41,530.8	0.3	− 20,733.4
DD ~ s(T) + s(Ws) + Thermo + Cs + Dgroup	30	41,535.0	3.9	− 20,737.5
DD ~ s(T) + s(Ws) + Cs + Dgroup	29	41,530.7	0.4	− 20,736.3
**DD ~ s(T) + s(Ws) + Cs**	**28**	**41,531.1**	**0.0**	− **20,737.6**
DD ~ s(T) + s(Ws)	27	41,535.9	4.8	− 20,740.9
DD ~ s(Ws)	25	41,565.8	34.7	− 20,757.9
DD ~ s(T)	25	41,705.9	174.8	− 20,828.0

In bold is the final selected model.

*T* temperature, *Ws* wind speed, *TL* total length, *Thermo* thermocline (presence/absence), *Cs* capture site, *Dgroup* depth group.

## Discussion

The acoustic telemetry technique used in this study provided valuable information on the habitat use of the endangered *S. umbra* within one of the most ancient MPAs in the Mediterranean Sea. This knowledge shed important lights on the importance of MPAs and fishing regulation measures for the protection of this species, but it further exposes fin-scale movements within the RNMBC which are important to consider in future management actions to ensure the recovery of the *S. umbra* populations in the region.

### Spatial distribution of *S. umbra*

Our results suggest that the RNMCB is extensive enough to protect the home range of the local *S. umbra* population. The brown meagre is highly sedentary, presenting a high residence index (0.58–1.00) in the RNMBC, as also reported elsewhere^15,37^. Most studies on *S. umbra*, though, have been performed by underwater visual census^15,61^ or by passive acoustic techniques^38,62,63^, which do not allow accurate estimates of home range sizes. Estimating the home range of endangered species is of great importance considering that their vulnerability to fishing mortality depends on the location of their home range relative to the reserve boundaries. Only those individuals with their entire home range within the reserve boundaries will be fully protected from fishing^64,65^. In RNMBC, the estimated home range of *S. umbra* was less than 1 km^2^ (0.31–0.82 km^2^), which was small enough to be completely included within the boundaries of this MPA. These results agree with previous reports (0.42–0.73 km^2^^37^), suggesting that even small protected areas effectively protect this species.

In the RNMBC the population of *S. umbra* is discretely distributed in two main areas: part of the population inhabits the area close to Cap Rédéris, within the FPA, while the remaining individuals inhabit the area close to Cap Abeille, in the PPA. The segregation (or aggregation) of individuals from the same species might be motivated by social or habitat segregation^66^. Social segregation refers to the formation of different groups, for instance, by age, sex, and/or size^66–68^. Although this can be the case for the *S. umbra*, which seems to aggregate according to age^69^, the two main distribution centers at RNMCB, do not seem to reflect social segregation since all individuals were mature adults and their size did not significantly affect our results. Thus, we believe that the observed distribution of *S. umbra* was driven by habitat segregation. Although only separated by ca. 1.5 km, this relatively isolated distribution of the population might be explained by habitat availability and discontinuity at small space scales^18,31,44^. The *S. umbra* typically prefers rocky areas^15,70^, where they can hide in burrows and caves in the center of their home range and perform excursions in the surrounding area^37,69^. The presence of an intermediate zone of sand and seagrass meadows between the two distribution areas might therefore act as barriers limiting the movements of *S. umbra*, as observed for other species^49,71^. We cannot exclude, though, a possible bias in the results associated with the VR2 network design^22,72^, due to the lack of receivers in the intermediate zone. However, *S. umbra* often avoids seagrass meadows and sandy habitats^70^ and is rarely spotted here by local recreational diving clubs or during the regular monitoring programs of marine communities in RNMCB (Lenfant, pers comm), suggesting little effect of the VR2 network design.

Habitat continuity might also explain the overall higher HR and DD observed for the *S. umbra* captured in the FPA. Here, larger areas of continuous rocky habitat likely promoted large homing movements^65,73^, explaining the observed differences between individuals collected at different sites. However, we cannot exclude the effect of the human activities on the behavior of the brown meagre. For instance, boat engine noise has already been shown to significantly affect the behavior of several fish species^74–78^. This includes the brown meagre, which responds with longer and more frequent flight and hiding behavior under higher levels of boat noise^79^, potentially reducing their dispersion away from the center of their home range. The two distribution areas of *S. umbra* at the RNMCB are indeed under different anthropogenic pressures: those collected at the FPA are under a low boat noise effect since all human activities, including boat traffic, are restricted. In contrast, those inhabiting the PPA might be subjected to higher human-induced stress, especially in summer, during higher recreational activity. This hypothesis requires further investigation, but if confirmed, human activities in the PPA, might induce unnecessary energy loss that could affect their fitness^75,79^ and modifications in their movement patterns, with possible implications on their reproduction and foraging activities.

### Temporal variability of *S. umbra* movements

A significant temporal pattern of *S. umbra* movements was evident in our results. Their home range fluctuated seasonally with a peak in June, but they seem to perform greater daily distances in winter, through shorter, but more frequent movements. This temporal variability is likely associated with reproductive and foraging behavior.

The brown meagre form spawning aggregations between May and August^38,46,47,80^ which were likely reflected in the temporal variability of their movements. First, the increase of the HR in June matched the peak of their reproduction activity^46,80^. Therefore, the dispersal of individuals away from the center of their home range at this time of the year might reflect reproductive movements to increase the probability of encountering mates. This has been previously reported for other species, such as the white seabream^81^, the kelp bass^32^ and even other Sciaenidae species^13^. Second, despite the high site fidelity and the small areas occupied by *S. umbra*, some individuals still performed seasonal migrations to areas outside their home range. Four individuals performed short-term (1–3 days) excursions from the PPA to the FPA between May and early June. Two-way short time movements had been previously reported for several fish species, usually associated with migrations to spawning areas^82–84^, supporting the hypothesis of spawning-related movements. For two of those individuals, though, these excursions were performed few days after tagging, suggesting a possible effect of the surgical procedure. However, most studies revealed little or no effect of tagging on fish behavior^43,85^ and reported effects were often described as increasing lethargy or impacts on buoyancy regulation^86,87^. This contrasts with the relatively distant excursion observed for these individuals. Furthermore, the remaining two individuals, performed similar migrations nearly one year after tagging, disproving this hypothesis and suggesting intentional movements. Similar migration patterns, were also performed by the fish 460, collected in the FPA but resident in the PPA. This individual performed frequent short-term migrations between the two areas, between mid-May and mid-August, concurrent with their reproduction season, supporting the hypothesis of seasonal movements for spawning aggregations^88^. Third, if this is true, it is expected to observe a higher overlap of individual home ranges with a peak during their spawning season^64^, which was indeed observed in our results, between May and July. Finally, a previous study performed with the same *S. umbra* individuals^19^, revealed a seasonal bathymetric distribution that was hypothesized to be also related to their reproduction behavior. Here it was shown that some individuals constantly lived in shallow waters all year round (the SS group) while the remaining part of the population shifted from deep waters (up to 50 m depth) in the cold months to shallow waters during the reproduction season (the DS group), where they all aggregated. Our results support this hypothesis by showing that higher CA overlap occurred during the spawning season in shallow waters (5–20 m depth) within the FPA, where rocky structures, caves and burrows are common. Therefore, we provide evidence that the main area of *S. umbra* spawning aggregations is located within the FPA, where human disturbance is limited. However, this does not imply that spawning aggregations only occur in this area. The presence of an established population of mature individuals in the PPA, nearby Cap Abeille, from which only one individual performed frequent migrations to the FPA during the reproduction season (fish 460), suggested that this area might also be a hotspot for *S. umbra* spawning aggregations. These results are of paramount importance, since they reveal when and where critical periods of the *S. umbra* occur.

Assessing the distances performed by *S. umbra* individuals on a daily basis using data from passive acoustic telemetry is a challenging task. Here, daily distances (DD) were calculated based on the half-linear distance between the position of the receivers. If the core area of one particular individual was located on the border of the detection radius of two or more receivers, it was very likely that the technique used here would overestimate the daily distances of that particular individual. Nevertheless, despite this bias, we believe that this variable allows us to carry out a valuable qualitative description of the temporal pattern of the distances performed by *S. umbra*, independently of their HR. The results of DD were surprising in the sense that larger daily distances were not performed either by the individuals that performed longer excursions or by those with larger HR. The temporal variability of these movements further indicated that *S. umbra* swam larger distances during colder months instead of during the reproductive season (although small peaks of DD were registered during this period). This suggests that individuals performed numerous small movements within a small area, rather than long linear excursions to areas far from their territory. A possible explanation for these results is likely associated with their foraging activity. Several studies reported an increase in voracity and predation activity of the brown meagre during the winter and a sharp slowdown in food intake during the summer^89–91^. The peaks of foraging activity appear to occur during the months that precede the active reproductive season, i.e. between February and April^46^, which matched with the observed peak of DD in our study. Likewise, the period of the year with lower estimated DD (August to October) corresponded to the resting post-spawning season, when individuals are suggested to remain less active in the protection of their caves^37,46^.

The temporal variability of *S. umbra* movements was also affected by environmental conditions. Our results highlight the role of strong winds in limiting their HR and DD. Several studies have identified the role of winds as the dominant mechanisms for dispersing fluvial plumes and resuspending sediments, reducing water visibility and increasing surface currents^92,93^. It is possible that *S. umbra* individuals reduce their homing movements and daily distances in order to remain close to or within their shelters, reducing the risks of predation and/or injury or reducing the probability of gamete dispersion during spawning, as also observed for other species^24,94^. However, strong wind conditions are known to reduce the detection probability of acoustic signals^22^, which might have affected the results. The negative effect of temperature on DD is likely indirect since the temperature is known to control the seasonal cycle of the *S. umbra* reproduction and foraging activity^37,46^, which likely controlled the observed temporal variability of *S. umbra* movements at the RNMBC (see above).

### Potential for spillover and connectivity

The spillover effect is an important benefit of MPAs, especially for local small-scale fisheries near the MPA borders^8,18,40,41,95^. However, from a conservation perspective, spillover of endangered species from an MPA increases their vulnerability to fisheries (legal or illegal), which might offset the effect of the protection measures on the recovery of their populations at larger spatial scales^5,7^. For *S. umbra*, their very high site fidelity and small HR, entirely located within the protected area, indicated a low potential for spillover, as suggested by Harmelin-Vivien et al.^15^ and García-Rubies^96^. However, spillover is still possible for *S. umbra*, particularly, nearby the MPA borders^18^, which might be facilitated by density-dependent movements and seasonal or ontogenic migrations^7,40,96,97^. Density-dependent movements typically refer to the emigration of individuals in response to the local density of conspecifics^7^. This type of fish migrations usually occurs when the biomass of the population attains the carrying capacity of the inhabited area, i.e. when the availability of resources becomes too low to support the local population^7,97^. As a result, some individuals may relocate their home range rather than endure food or space-limited conditions. This might be the case of the *S. umbra* in the RNMBC. The presence of an established group of *S. umbra* individuals in the PPA, nearby Cap Abeille, might be seen as an effect of spillover from the FPA. Indeed, monitoring programs of the abundance of endangered species in RNMCB indicate an increase of the *S. umbra* abundance, particularly in the PPA, where the rate of recovery is 4 times higher than in the FPA (an increase of 19.9 vs. 4.8 ind. year^−1^, respectively, between 2011 and 2020^98^). This seems to be also the case of *S. umbra* populations inhabiting another ancient Mediterranean MPA (Medes Islands Marine Reserve in Spain, created in 1983), where the population also stabilized^96,99^. Considering the advanced age of the RNMBC^10^, this might suggest that high levels of *S. umbra* density have achieved or are close to the carrying capacity in the FPA, which, coupled with erratic dispersal behaviour, induced juvenile and/or adult individuals to seek available habitat niche^97^, leading to the establishment of satellite population in the nearby rocky areas of the PPA (i.e. close to Cap Abeille). This might be perceived as a positive effect of the protection measures in the recovery of the local *S. umbra* populations, sustaining high levels of spillover to partially- and, possibly, to non-protected areas.

The export of fish biomass from MPAs also results from the migration of individuals beyond its boundaries^7,97^. Despite their highly sedentary behavior, some individuals did migrate beyond the borders of RNMBC. During our study period, three individuals left the detection array. One of them, captured within the FPA, travelled to a Spanish MPA (Cap de Creus Natural Park), covering a distance of at least 21 km from their collection site. Although this type of excursion was only recorded for one individual, it is of particular importance. First, it supports the existence, although restricted, of *S. umbra spillover* effect from the RNMBC. Second, it provides evidence that long-distance migrations might contribute to the exchange of individuals to other protected areas. This would help to explain the successful recovery of their populations in MPAs, despite the scarce records of juveniles in these areas^96^. Finally, this might have important implications for the recovery of the population at larger time and space scales, since long distance migrations of adult individuals have an important role in the connectivity between isolated populations^100,101^.

## Conclusion and management implications

The brown meagre *S. umbra* is a very desired and easy target for fisheries (especially spearfishing), due to its peaceful behavior, gregarious character, accessibility and gastronomic value^15–17^, which highlight its vulnerability. The decline of their populations in the Mediterranean Sea, with estimated reductions of 70% between 1980 and 2005^102^, uncovered the need for specific protection measures. Among these measures, MPAs are considered one of the most efficient ways to support the recovery of their population due to the simultaneous protection of species and their habitat^15,96^. This is supported by our results, which give evidence that the RNMCB, although small, is still large enough to provide effective protection for *S. umbra* by covering its home range and its main spawning aggregation areas. However, the sole protection of the species within MPAs might help, but it does not guarantee the recovery of the populations at larger time and spatial scales. Therefore, the reinforcement and perpetuation of protection measures are essential to ensure the recovery of the population inside and outside MPAs. In this sense, the combination of spatial protection measures, such as the creation and expansion of MPAs, and fisheries management, such as a ban on fishing for endangered species, might largely promote the conservation and rate of recovery of *S. umbra* populations. These measures are currently being applied in France, with the expansion process of the RNMCB and the actual moratorium for *Sciaena umbra* and *Epinephelus marginatus*, but it should be perpetuated and extended to all Mediterranean countries.

Here we provide evidence that the population of *S. umbra* is not limited to the fully protected area, and density-dependent movements likely contributed to the establishment of a satellite population nearby Cap Abeille. We further show that this area might be an important spawning area for *S. umbra*. This should be considered in local conservation plans. The area around Cap Abeille is at the PPA and, therefore, exposed to higher levels of human-induced stress, such as boat noise. Considering the sensitivity of *S. umbra* to anthropogenic noises, a full reduction of the human impact on those areas could largely improve the efficiency of *S. umbra* reproduction. This would be of great significance during the spawning aggregations, which peak in June and at dusk hours^19^. The expansion of both areas of the RNMBC (FPA and PPA) would not only promote the conservation of *S. umbra*, but all its habitats and the associated community, increasing the local biodiversity conservation ^2^. In France, this is indeed the objective of the new National Strategy for Marine Protected Areas (https://www.ofb.gouv.fr/la-strategie-nationale-pour-les-aires-protegees), which announced the ambition to protect 30% of the national marine and terrestrial areas by 2030, including 10% in no-take zones.

Furthermore, our study supports the need to perpetuate the French moratorium in place until 2023, and establish it in other Mediterranean countries. Our results show that although restricted, short seasonal migrations to spawning areas and long-distance excursions beyond RNMBC boarders of *S. umbra* are occurring and possible more frequent than previously thought, even extending to other neighbor MPAs such as Cap de Creus in Spain. These movements are critical if we want to witness the recovery of *S. umbra* populations at a larger spatial scale since they are fundamental to keep the connectivity among the Mediterranean populations. However, these movements also highlight their vulnerability to fishing outside MPAs, especially in border areas with different fishing regulations, as in this case study (*S. umbra* fishing ban in France but absent in Spain). This supports the need to ensure their protection at larger spatial scales, as proposed by the French moratoria, and should therefore be maintained and extended to the other Mediterranean countries. However, these measures are useless if not accompanied by surveillance efforts. Small MPAs, like RNMCB, might have enough resources for local actions of control and inspection of the activities within their borders, but this becomes a challenging task at larger spatial scales. The creation of the Gulf of Lion Marine Natural Park in 2011 (https://www.parc-marin-golfe-lion.fr/) aims to overcome this problem and ensure a coherent and non-fragmented management of a large maritime area. Overall, our study shows the importance of these conservation measures for the protection of *S. umbra*, but it further supports the need to perpetuate and reinforce them to ensure the successful recovery of the *S. umbra* populations in the Mediterranean Sea.

## Data Availability

The datasets generated and analysed during the current study are available from the corresponding author on reasonable request.
